# Supplementary material for: Reprogrammed CD8+ T-Lymphocytes Isolated from Bone Marrow Have Anticancer Potential in Lung Cancer
Source: Biomedicines. 2022 Jun 19;10(6):1450. doi: 10.3390/biomedicines10061450 (PMC9219954; doi:10.3390/biomedicines10061450)
Supplement: Supplementary file 1 [file biomedicines-10-01450-s001.zip › biomedicines-1746982-supplementary.pdf]

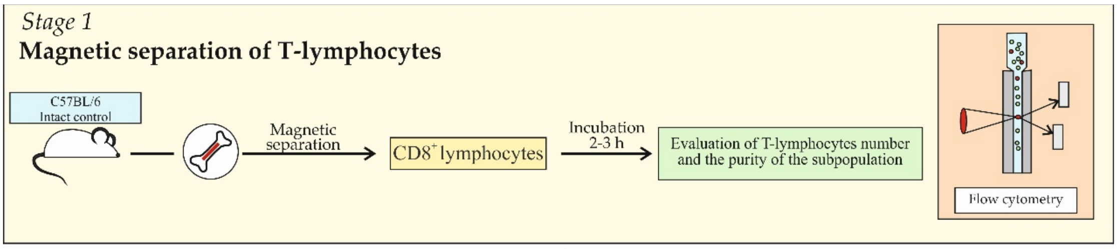

(a)

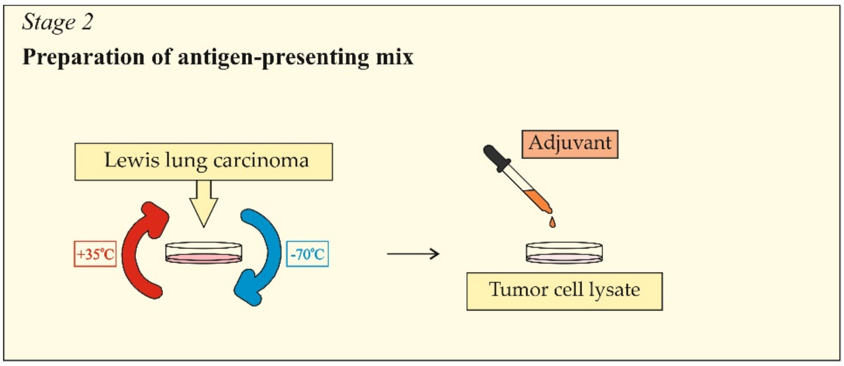

(b)

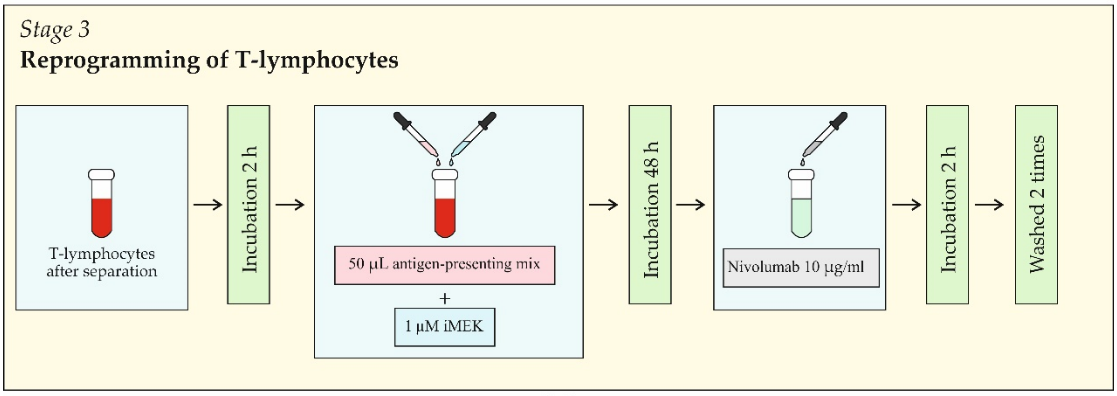

(c)

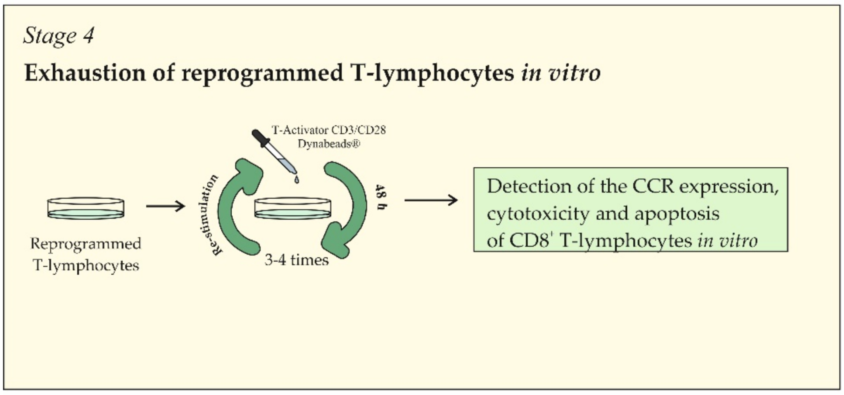

(d)

Figure S1: Main stages of the T-lumphocytes reprogramming. **(a)** T-lymphocytes isolated from the bone marrow of C57/BL6 mice. The cell suspension was enriched with $CD8^{+}$ T-lymphocytes using magnetic separation. the number of cells and the purity of the population were evaluated by flow cytometry after separation. **(b)** An antigen-presenting mix was prepared. The antigen-presenting mix was a LLC lysate consisting of a mix of tumor antigens. To increase the immune reaction, an adjuvant

was added to the LLC lysate. **(c)** For reprogramming, 50 μL of an antigen-presenting mix with 1 μM MEK inhibitor was added to a flask with $CD8^+$ T-lymphocytes. The resulting cell suspension was incubated for 48 h. Reprogrammed $CD8^+$ T-lymphocytes were incubated for 2 h with human monoclonal antibody (MAT) nivolumab (10 μg/ml). At the end of the incubation cycle, suspensions were washed 2 times in the medium recommended for T-lymphocytes. **(d)** To estimate the population stability, the exhaustion of reprogrammed T-lymphocytes was carried out *in vitro*. Reprogrammed T-lymphocytes in the medium supplemented with IL-2 (30 IU/ml) were stimulated by T-Activator CD3/CD28. Re-stimulation was performed 3-4 times every 48 hours. Then, the Dynabeads were removed. Immunophenotype and cytotoxicity of reprogrammed T-lymphocytes were analyzed.
